# *TMEM14A* Gene Affects Hippocampal Sclerosis in Mesial Temporal Lobe Epilepsy

**DOI:** 10.3390/jcm14113810

**Published:** 2025-05-29

**Authors:** Joonho Kim, Soomi Cho, Kyoung Hoon Jeong, Woo-Seok Ha, Kyung Min Kim, Min Kyung Chu, Ji Hyun Lee, Sangwoo Kim, Won-Joo Kim

**Affiliations:** 1 Department of Neurology, Yonsei University College of Medicine, Seoul 03722, Republic of Korea; joonho345@yuhs.ac (J.K.); soomicho@yuhs.ac (S.C.); chumk@yuhs.ac (M.K.C.); 2 Department of Biomedical Systems Informatics, Brain Korea 21 PLUS Project for Medical Science, Yonsei University College of Medicine, Seoul 03722, Republic of Korea; swkim@yuhs.ac; 3 Epilepsy Research Institute, Yonsei University College of Medicine, Seoul 03722, Republic of Korea; jeongkh81@yuhs.ac; 4 Department of Clinical Pharmacology and Therapeutics, Kyung Hee University College of Medicine, Seoul 02447, Republic of Korea; hyunihyuni@khu.ac.kr

**Keywords:** single-nucleotide polymorphisms, transcriptomics, splicing quantitative trait locus analysis, *TMEM14A*

## Abstract

**Background**: Hippocampal sclerosis (HS) is a hallmark of mesial temporal lobe epilepsy (MTLE). However, genetic studies on MTLE patients with HS (MTLE-HS) remain limited, especially in East Asian populations. This study aimed to identify genetic variants associated with MTLE-HS and elucidate their biological relevance through integrative genomic and transcriptomic analyses. **Methods**: We conducted a genome-wide association study (GWAS) on 157 Korean epilepsy patients, including 52 MTLE-HS subjects and 105 non-acquired focal epilepsy individuals without HS as controls. The splicing and expression quantitative trait locus (sQTL and eQTL, respectively) effects of significant variants were analyzed using GTEx datasets. Transcriptomic data from the hippocampi of MTLE-HS subjects and an epilepsy mouse model were examined to assess *TMEM14A* expression. Gene correlation enrichment analysis was performed to investigate potential associations with epilepsy-related phenotypes. **Results**: The GWAS identified rs6924849, located downstream of *TMEM14A*, as significantly associated with MTLE-HS. The sQTL analysis revealed that rs6924849 induces abnormal *TMEM14A* splicing in hippocampal tissue. Transcriptomic analyses showed reduced *TMEM14A* expression in MTLE-HS hippocampi, while mice with pilocarpine-induced epilepsy exhibited a transient increase in *TMEM14A* expression during the acute phase post-status epilepticus. Gene correlation enrichment analyses linked *TMEM14A* to seizure-related phenotypes in both humans and mice. **Conclusions**: This study identifies rs6924849 as a novel genetic variant associated with MTLE-HS in an East Asian population. The dysfunctional splicing and altered expression of *TMEM14A* may contribute to the neuronal loss characteristic of HS, as *TMEM14A* regulates apoptosis. These findings emphasize the potential role of *TMEM14A* in MTLE-HS pathogenesis from genomic and transcriptomic perspectives.

## 1. Introduction

Epilepsy, one of the most prevalent and debilitating neurological disorders, has a well-recognized genetic component [1]. Historically, the predominant focus in genetic studies has been on the Mendelian forms of epilepsy, such as developmental and epileptic encephalopathies (DEEs). Genome-wide association studies (GWASs) have recently been used to investigate the genetic component of common forms of epilepsy such as genetic generalized epilepsy (GGE) and non-acquired focal epilepsy. These efforts have resulted in the discovery of numerous genetic loci associated with epilepsy, notably in large-scale studies [2,3,4]. Despite these advancements, research on mesial temporal lobe epilepsy with hippocampal sclerosis (MTLE-HS), one of the most refractory forms of epilepsy, has not resulted in definitive outcomes.

MTLE-HS represents a unique clinical and pathological subtype of epilepsy, often requiring surgical intervention due to its resistance to medical therapy [5]. Initial precipitating injuries, such as febrile convulsions, have been linked to the development of HS [6]. However, only a subset of individuals with such injuries progresses to MTLE-HS, indicating a likely interplay between genetic predisposition and environmental factors. In previous GWASs on MTLE-HS, such as the study by Kasperaviciute et al., genetic loci were identified including the SCN1A region [2,3,4,7]. However, the findings specific to MTLE-HS have not been consistently replicated in subsequent studies, and loci around the SCN1A region have instead been associated more broadly with all epilepsy, focal epilepsy, or GGE. This inconsistency is likely attributable to the use of general population controls rather than epilepsy-specific controls, which may obscure the genetic signals that are uniquely associated with MTLE-HS.

In addition, genetic studies on MTLE-HS have predominantly focused on European populations, leaving the East Asian population largely underrepresented. Given the differences in genetic architecture between populations of different ancestries, studies targeting East Asian cohorts are critical for uncovering population-specific genetic risk factors [8]. This gap underscores the need for an East Asian-focused investigation of MTLE-HS.

In this study, we address these limitations by performing a GWAS of MTLE-HS in an East Asian population, utilizing non-acquired focal epilepsy patients without HS as controls to minimize confounding factors. We further explore the biological relevance of a genetic association through integrative analyses, including splicing and expression quantitative trait locus (sQTL and eQTL, respectively) studies, as well as transcriptomic analyses in human MTLE-HS subjects and an epilepsy mouse model. The findings provide insights into the pathogenesis of MTLE-HS and highlight the potential therapeutic targets for this challenging condition.

## 2. Materials and Methods

### 2.1. Subjects

This study collected blood samples and obtained genotype data from 157 individuals with focal epilepsy who were recruited from the epilepsy clinic at Severance Hospital, Yonsei University College of Medicine, between June 2016 and November 2018. All participants were of Korean ancestry, and familial relationships among them were excluded. Among the participants, 52 were diagnosed with HS (27 females and 25 males); 105 without HS (48 females and 57 males) were included as the control group (Table 1). Experienced epileptologists diagnosed focal epilepsy based on clinical manifestations or electroencephalographic findings following the seizure classification criteria established by the International League Against Epilepsy [9]. The presence of HS was assessed using brain magnetic resonance imaging and was independently evaluated by radiologists and epileptologists. Individuals with progressive epilepsy syndromes or epileptic encephalopathies were excluded from both groups, leaving only those with non-acquired focal epilepsy. Statistical analyses for comparing clinical characteristics were conducted with chi-square tests, Fisher’s exact tests, and Student’s *t*-tests using R (version 4.2.0).

### 2.2. Study Approval

Written informed consent for genetic analysis was obtained from each participant or their legally authorized representative after a thorough explanation of the study details. This research was conducted in accordance with the principles outlined in the Declaration of Helsinki and received approval from the Institutional Review Board (IRB) of Gangnam Severance Hospital, Yonsei University College of Medicine, Seoul, South Korea (IRB No. 3-2016-0096).

### 2.3. Genotyping and Quality Control of SNPs

Genotyping was performed in 2019 using the GeneChip Human Mapping 500K Array Set (Affymetrix) in accordance with the manufacturer’s standard protocol. The quality control of the samples and SNPs adhered to established guidelines detailed in previous research [10,11,12]. Sample quality control criteria included the following: population stratification principal component analysis scores within ±6 standard deviations, genotype call rate > 96%, concordance between estimated and reported sex, and no evidence of excessive heterozygosity (>mean + 3 standard deviations or <mean − 3 standard deviations). Population stratification was evaluated through PCA using SmartPCA (EIGENSOFT) software (version 6.1.4) and reference data from HapMap Phase III populations, including European (CEU), African (YRI), Japanese (JPT), and Han Chinese (CHB) [13]. Sex estimation was calculated using the mean homozygosity rate across the X chromosome.

The SNP quality control criteria required a minor allele frequency (MAF) > 0.01, genotype call rate > 95% in both groups, and Hardy–Weinberg equilibrium with a *p*-value > 1 × 10^−4^ in the control group. A total of 440,398 SNPs were initially analyzed, including 430,623 autosomal SNPs, 155 SNPs in the pseudo-autosomal region of the X chromosome, and 9620 SNPs on the X chromosome. Following quality control, 290,091 SNPs remained for further analysis.

### 2.4. Genome-Wide Association Study

A GWAS was conducted using an additive model and logistic regression analysis implemented in PLINK (version 1.9) because all participants were of Korean descent and sampled from a single center without batch effects [14]. Each SNP was annotated using the Affymetrix gene annotation database and the UCSC Genome Browser. A quantile–quantile plot and Manhattan plot were generated with the GWAS results using PLINK (version 1.9).

### 2.5. Allele Frequency in Normal Population

Allele frequencies for the Korean, Japanese, and European populations were obtained from the Korea Centers for Disease Control and Prevention (KCDC), 14K JPN, and the 1000 Genomes Project Phase 3 in the Database of Single Nucleotide Polymorphisms (dbSNP), respectively (https://www.ncbi.nlm.nih.gov/snp/, accessed on 1 October 2024).

### 2.6. Genetic Correlation Analysis

We performed LDSC (version 1.0.1) and popcorn (version 1.0.0) to estimate the genetic correlations for within-EAS and cross-ancestry genetic correlations between the EAS and EUR populations [15]. The LD scores of the EAS and EUR populations from 1000 Genomes were obtained from Google cloud provided by LDSC (console.cloud.google.com/storage/browser/broad-alkesgroup-public-requester-pays, accessed on 1 October 2024). We filtered the SNPs using the HapMap3 SNPs provided by LDSC.

### 2.7. Phenome-Wide Association Study

A phenome-wide association study (PheWAS) was performed for candidate SNPs, and Manhattan plots were generated using the UKBiobank ICD PheWeb platform (https://pheweb.org/UKB-SAIGE/, accessed on 1 October 2024).

### 2.8. Quantitative Trait Locus Analyses

The sQTL and eQTL for the rs6924849 variant were investigated using the GTEx version 8 dataset. The QTL parquets files across 47 tissues and a violin plot of normalized intron excision ratios for rs6924849 were retrieved from the GTEx Portal (https://gtexportal.org/home/, accessed on 4 June 2024). The pipeline of preprocessing, expression quantification, and QTL analysis is thoroughly described in “Analysis Methods” on the GTEx Portal. For QTL analysis, cis-eQTL and cis-sQTL mapping were conducted using FastQTL, defining a 1-megabase window around transcription start sites [16]. Normalized effect size (NES), representing the effects of the alternative allele relative to the reference allele, was defined as the slope of the linear regression and calculated in a normalized space. LeafCutter was used to quantify splicing using intron excision phenotypes [17].

### 2.9. TMEM14A Expression Across Bulk Tissues

The violin plot for *TMEM14A* expression levels across bulk tissues was obtained from the GTEx Portal on (https://gtexportal.org/home/, accessed on 4 June 2024). Expression levels were normalized using transcripts per kilobase million (TPM).

### 2.10. Transcriptomic Analysis in MTLE with HS

We collected transcriptomic data from seven studies on MTLE with HS, all of which provided publicly available RNA sequencing data in the Sequence Read Archive (SRA) [18,19,20,21,22,23,24]. Only hippocampal RNA data from MTLE-HS and normal controls were included, and data from other brain regions, such as the cortex, were excluded. The dataset comprised RNA sequencing data from 142 MTLE-HS patients and 134 normal controls.

The RNA data were filtered and trimmed using FASTP (version 0.21.0), and the filtered RNA reads were aligned to the human genome reference (GRCh38.p14) using STAR (version 2.7.10) [25,26]. Transcript counts for each gene were quantified using HTSeq (version 2.7.17) [27]. To address batch effects across the seven cohorts, combat-seq (from the sva package, version 3.46.0) was applied with MTLE and normal controls included as covariates [28]. Transcript counts were normalized to TPM using GeoTcgaData (version 1.99.2) [29]. Finally, normalized *TMEM14A* and *ACTB* expression levels were compared between MTLE-HS subjects and normal controls using Student’s *t*-tests in R (version 4.2.0).

### 2.11. Transcriptomic Analysis in an Epilepsy Mouse Model

The hippocampal RNA sequencing data of a pilocarpine-induced epilepsy mouse model were obtained from the study by Popova et al. and are accessible on the Sequence Read Archive [30]. Hippocampal tissues from pilocarpine-induced epilepsy mice were extracted at the following post-treatment time points: 1 h (*n* = 3), 8 h (*n* = 2), 36 h (*n* = 3), and 120 h (*n* = 3). For normal control mice treated with saline instead of pilocarpine, hippocampal tissues were extracted at 1 h (*n* = 2), 8 h (*n* = 3), and between 36 and 120 h (*n* = 3).

The same filtering, alignment, and quantification pipeline used for human data was applied here, except for aligning reads to the mouse genome reference (GRCm39). Differential gene expression was analyzed using DESeq2 (version 1.38.3) at each time point, and the results were expressed as log2 fold changes [31]. For differential expression analysis, the hippocampal samples of normal control mice extracted between 36 and 120 h were compared with either 36 h or 120 h samples from pilocarpine-induced epilepsy mice.

### 2.12. Prediction of Human and Mouse Phenotypes

The human and mouse phenotype gene sets associated with *TMEM14A* were retrieved from the All RNA-seq and ChIP-seq Sample and Signature Search (ARCHS4) database (https://maayanlab.cloud/archs4, accessed on 10 November 2024). ARCHS4 integrates all publicly available human and mouse RNA-seq experiments from the Gene Expression Omnibus (GEO) and the Sequence Read Archive (SRA) to predict associations between gene sets across various biological domains and the gene of interest [32]. The predictions are based on Z-scores, which quantify the correlation between *TMEM14A* and the known members of each gene set. A higher Z-score indicates a stronger correlation and a higher likelihood that *TMEM14A* is a member of the corresponding gene set.

### 2.13. Data Availability

The summary statistics for all SNPs included in this GWAS is available through the Figshare repository with the following digital object identifier (DOI): https://doi.org/10.6084/m9.figshare.27948009.v1. Japanese GWAS summary statistics are available at Biobank Japan with phenotype “Epilepsy” (https://pheweb.jp/pheno/Epilepsy, accessed on 15 September 2024), and European and trans-ethnic GWAS summary statistics are available in the NHGRI-EBI GWAS Catalog under the following accession numbers: GCST90271608, GCST90271611, GCST90271612, GCST90271613, GCST90271618, GCST90271619, and GCST90271620 [4,33]. Raw RNA sequencing data for MTLE-HS are available at Sequence Read Archive under the following accession numbers: PRJNA290212, PRJNA373909, PRJNA525671, PRJNA556159, PRJNA1073977, PRJNA280563, and PRJNA1077986 [18,19,20,21,22,23,24]. In addition, raw RNA sequencing data for the pilocarpine-induced epilepsy mouse model are available under the accession number PRJNA815649 [30]. sQTL and eQTL GTEx data (version 8) across 47 tissues can be accessed through the GTEx Portal website (https://gtexportal.org/home/, accessed on 4 June 2024).

## 3. Results

### 3.1. Genome-Wide Association Study

All individuals included in the analysis successfully met the rigorous quality control criteria detailed in the Section 2. A chi-square test confirmed no significant sex differences between the case and control groups (*p* = 0.573; Table 1). The quantile–quantile plot of observed versus expected *p*-values showed adherence to quality control standards and provided evidence for potential associations (Figure S1). Among the 440,398 initial SNPs, 290,091 remained for downstream association analysis after quality filtering.

**Figure 1:**
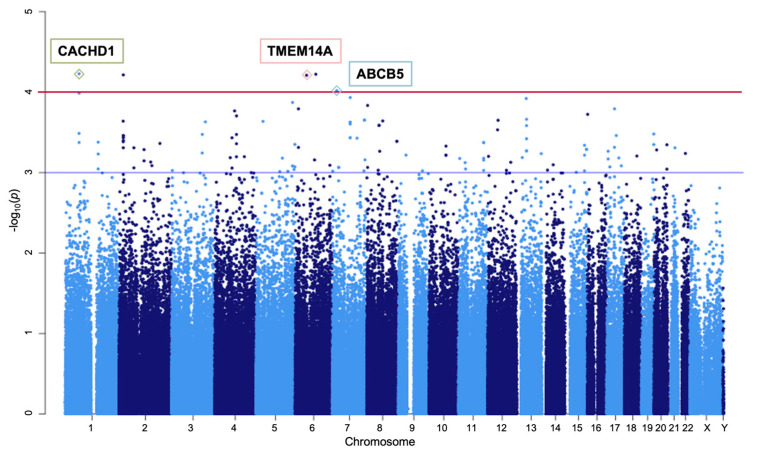
A Manhattan plot of genotype data. The plot displays 290,091 SNPs based on their chromosomal positions. The −log10(*p*-value) was derived from an allelic association test. Potential candidate SNPs are marked with the following colors: green for *CACHD1*, red for *TMEM14A*, and blue for *ABCB5*. The red horizontal line represents the significance threshold for association analysis (*p* = 1.0 × 10^−4^), while the blue line marks *p* = 1.0 × 10^−3^. Abbreviation: SNP, single-nucleotide polymorphism.

The Manhattan plot of the GWAS results revealed an overview of SNP-level associations (Figure 1). A total of 127 SNPs exhibited a *p*-value < 1 × 10^−3^ (Table S1). Five candidate SNPs met the criteria of *p* < 1 × 10^−4^ and independent loci (Table 2). Among these, rs6924849, located downstream of *TMEM14A*, showed an MAF of 0.4423 in MTLE-HS cases compared with 0.1857 in controls, yielding an odds ratio of 2.685.

### 3.2. Phenome-Wide Association Study

A PheWAS was conducted for the five candidate SNPs using the UK Biobank database. The analysis identified a significant association between rs6924849 and the phenotype “Other conditions of brain, NOS” (*p* = 1.10 × 10^−4^), indicating a potential link to neurological disorders. Notably, the other candidate SNPs did not exhibit significant associations with neurological phenotypes (Figure S2).

### 3.3. Comparative Genetic Architecture Across Ancestries and Subtypes

**Figure 2:**
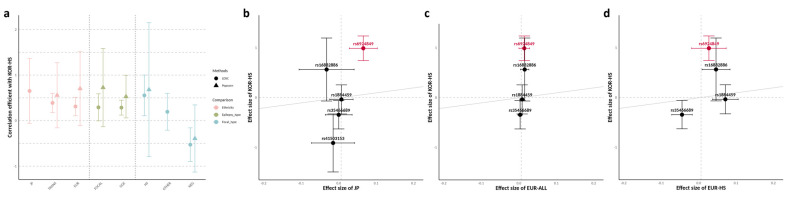
A comparison of genetic architecture across ancestries and subtypes from external cohorts. (**a**) The SNP-based genetic correlation values (rg) of the GWAS results from this study across multiple ethnic groups, epilepsy types, and focal epilepsy subtypes. Each point represents the genetic correlation estimated by LDSC (circle) and popcorn (triangle), with error bars indicating the 95% confidence intervals. The target populations of correlation analysis are color-coded: red for ethnicity, green for epilepsy types in European GWAS data, and blue for focal epilepsy subtypes in European GWAS data. TRANS indicates trans-ethnic GWAS data including the East Asian population. Popcorn estimates for JP and OTHER are excluded as their values fall outside the correlation coefficient range (−1.0 to 1.0). (**b**–**d**) Scatter plots showing the effect sizes (BETA) of variants annotated with *TMEM14A*, including rs6924849, the most significant variant in this study (highlighted in red). The Y-axis represents the effect size from this study, and the X-axis represents the effect size of the following data: (**b**) the BBJ GWAS data of all epilepsy; (**c**) the European GWAS data of all epilepsy; (**d**) the European GWAS data of focal epilepsy with HS. rs41503153 was not included in the European GWAS. Japanese GWAS data were extracted from BBJ with phenotype “Epilepsy”, while European and trans-ethnic GWAS data were extracted from Stevelink et al., 2023 [4]. Abbreviations: KOR, Korean; HS, focal epilepsy with hippocampal sclerosis; JP, Japanese; TRANS, trans-ethnic; EUR, European; FOCAL, focal epilepsy; GGE, genetic generalized epilepsy; OTHER, focal epilepsy with other lesions; NEG, focal epilepsy without lesions; SNP, single-nucleotide polymorphism; GWAS, genome-wide association study; BBJ, Biobank Japan.

Genome-wide SNP-based genetic correlation analysis for all epilepsy subtypes revealed similar estimates across the Japanese, trans-ethnic, and European populations (Figure 2a). However, a noticeable discrepancy in allele frequencies was observed between the East Asian and European populations for the identified candidate SNPs, with the Korean and Japanese populations showing comparable allele frequencies (Table 2). Specifically, the allele frequency of rs6924849 was markedly higher in the East Asian populations (0.1938 in Koreans, 0.1923 in Japanese) compared with Europeans (0.0467), indicating a stronger polymorphism in East Asians. The effect sizes of SNPs surrounding *TMEM14A*, including rs6924849, in the Japanese population exhibited trends relatively consistent with our findings compared with those in the European population (Figure 2b,c).

The genetic correlations with focal epilepsy and GGE showed comparable estimates, likely due to the use of focal epilepsy without HS as the control group, which effectively minimized confounding effects from general focal epilepsy (Figure 2a). The genetic correlation data also revealed a stronger correlation with HS in European datasets compared with focal epilepsy with other lesions or focal epilepsy without lesions. Notably, focal epilepsy without lesions exhibited a negative correlation, likely because most of the control group consisted of non-lesional focal epilepsy cases with unknown etiology (Table 1). However, a clear discrepancy in effect sizes for SNPs surrounding *TMEM14A*, including rs6924849, was observed between MTLE-HS in the Korean and European populations (Figure 2d).

### 3.4. Quantitative Trait Locus Analysis for rs6924849

**Figure 3:**
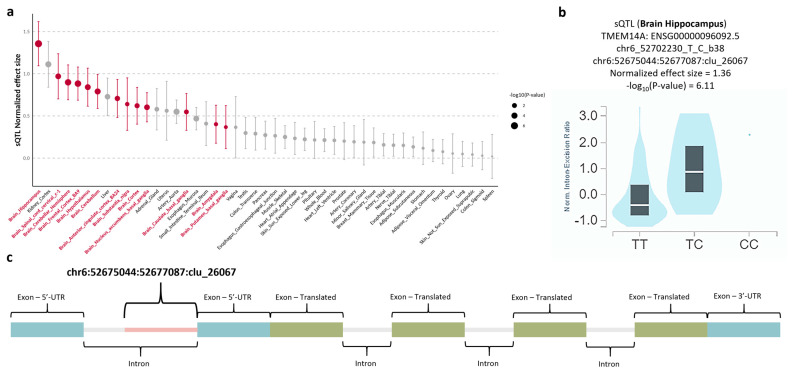
Splicing quantitative trait locus (sQTL) analysis for rs6924849. (**a**) The normalized sQTL effect size of rs6924849 across 47 tissues from the GTEx v8 dataset, with brain and non-brain tissues highlighted in red and grey, respectively. The −log10(*p*-value) is represented by the size of each point. Error bars indicate the 95% confidence intervals of the effect size. (**b**) The *TMEM14A* normalized intron excision ratio by genotype for rs6924849 in hippocampal tissue. The central line in the box plot represents the median (50th percentile), while the box edges correspond to the 25th and 75th percentiles (interquartile range). The splicing cluster identifier is chr6:52675044:52677087:clu_26067. This violin plot was obtained from the GTEx portal website. (**c**) The location of the splicing cluster chr6:52675044:52677087:clu_26067 within the *TMEM14A* gene. The regions are annotated as follows: untranslated regions (UTRs) in blue, translated regions in green, introns in gray, and chr6:52675044:52677087:clu_26067 in red. Abbreviations: GTEx, Genotype-Tissue Expression; sQTL, splicing quantitative trait loci; UTR, untranslated region.

To elucidate the regulatory effects of rs6924849 on *TMEM14A*, we performed sQTL and eQTL analyses using the GTEx version 8 dataset. The sQTL analysis identified significant abnormal splicing events in hippocampal tissue, followed by other brain regions (Figure 3a). Specifically, the presence of the rs6924849 variant (T → C) was associated with an increased intron excision ratio in the hippocampus, corresponding to the splicing cluster chr6:52675044:52677087:clu_26067 (Figure 3b). This intronic region, located between two exons of the 5′-UTR, is normally spliced out; however, the variant appears to impede this process, resulting in aberrant transcripts (Figure 3c). In contrast, the eQTL analysis revealed only modest changes in *TMEM14A* RNA expression levels across various tissues (Figure S3).

### 3.5. Transcriptomic Investigation for TMEM14A

**Figure 4:**
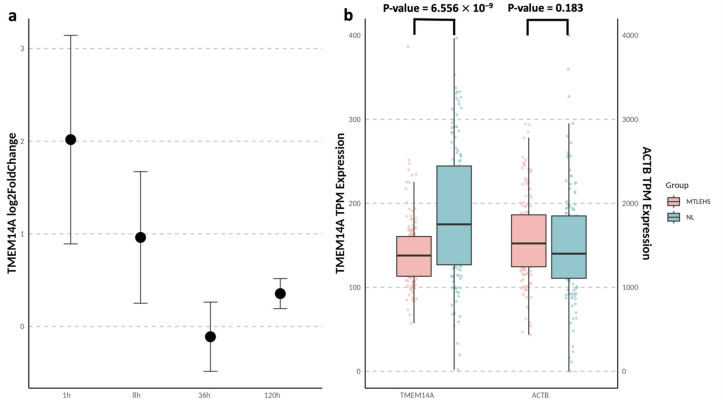
*TMEM14A* expression in MTLE-HS patients and pilocarpine-induced epilepsy model. (**a**) *TMEM14A* hippocampal differential expression (log2FoldChange) of pilocarpine mouse model compared to normal controls at different post-treatment time points (1 h, 8 h, 36 h, and 120 h). Error bars indicate the 95% confidence intervals of the log2FoldChange. (**b**) *TMEM14A* hippocampal gene expression (TPM) in MTLE-HS and NL. Statistical significance was assessed using a two-sided *t*-test, with the *p*-value provided. The central line in the box plot represents the median (50th percentile), while the box edges correspond to the 25th and 75th percentiles (interquartile range). All human and mouse gene expression data were obtained from the Gene Expression Omnibus (GEO) database. Abbreviations: MTLE, mesial temporal lobe epilepsy; HS, hippocampal sclerosis; NL, normal controls; TPM, transcripts per kilobase million.

In a pilocarpine-induced epileptic mouse model, *TMEM14A* expression exhibited a rapid and transient increase during the acute phase (1 and 8 h post-treatment), followed by stabilization during the chronic phase (36 and 120 h) (Figure 4a). Conversely, *TMEM14A* expression was significantly reduced in hippocampal tissue from patients with chronic MTLE-HS compared with normal controls, with *ACTB* serving as the housekeeping gene showing consistent expression between the two groups (Figure 4b). *TMEM14A* expression was enriched across all brain regions in normal individuals compared with other tissues based on the GTEx version 8 dataset (Figure S4).

A gene correlation enrichment analysis of human and mouse transcriptomic data revealed potential associations between *TMEM14A* and seizure-related phenotypes. Predicted human phenotypes included “Focal motor seizures” and “Focal seizures”, and mouse phenotypes included “Abnormal synaptic transmission” and “Seizures” (Table S2).

## 4. Discussion

To the best of our knowledge, this is the first study in which a GWAS of MTLE-HS was conducted in an East Asian population. Although the focus in previous studies including Chinese and Japanese cohorts has been on epilepsy in general, in the present study, MTLE-HS was specifically targeted [34,35]. Notably, in previous European GWASs, rs6924849 was not identified likely due to the low MAF of the variant in European populations. The observed disparities in genetic architecture underscore the necessity of population-specific GWASs to uncover genetic risk factors for MTLE-HS.

We aimed to minimize confounding factors related to general focal epilepsy and isolate genetic components specific to MTLE-HS using non-acquired focal epilepsy without HS as the control group. Genetic correlation analyses confirmed that confounding effects from general focal epilepsy were successfully mitigated. Notably, a negative genetic correlation was observed in focal epilepsy without lesions, likely attributable to the predominance of cryptogenic focal epilepsy without lesions in the control group. While this control selection may introduce some bias by excluding shared genetic features between MTLE-HS and other focal epilepsy subtypes, we considered that the genetic specificity toward MTLE-HS outweighs this limitation and enhances the suitability of this study for identifying significant variants specific to MTLE-HS.

We thoroughly investigated rs6924849 among the five candidate SNPs, as the PheWAS analysis indicated its potential association with neurological disorders. This variant, located in the non-coding region downstream of *TMEM14A*, was evaluated for its effect on gene regulation and found to significantly increase abnormal splicing events, particularly in hippocampal tissue. This disruption appears to hinder proper splicing, resulting in an abnormal transcript that retains this region and likely disrupts *TMEM14A* translation in the hippocampus considering the critical role of the 5′-UTR sequence in translation regulation. While sQTL analysis revealed a pronounced splicing alteration in the hippocampus, the eQTL effect across tissues was minimal, suggesting that this variant may primarily influence transcript structure rather than transcript abundance.

Following the analysis of rs6924849, we explored the role of *TMEM14A* and its potential association with epilepsy. *TMEM14A* regulates apoptosis by stabilizing mitochondrial membrane potential and preventing mitochondrial permeability transition [36]. However, the specific role of *TMEM14A* in brain tissue and neurological disorders remains poorly understood. In this study, gene correlation enrichment analysis revealed a potential link between *TMEM14A* and seizure-related phenotypes in both humans and mice. Furthermore, *TMEM14A* was highly expressed across most brain regions, indicating its importance in maintaining normal brain function.

To evaluate this association, *TMEM14A* expression was examined in human MTLE-HS subjects and in an animal epilepsy model. In a pilocarpine-induced epilepsy mouse model, *TMEM14A* expression showed a dramatic increase during the acute phase (1 and 8 h post-status epilepticus), likely reflecting a compensatory response to regulate the apoptosis of neurons. Considering the regulatory role of *TMEM14A*, its dysfunction may lead to unregulated apoptosis following neuronal damage, ultimately contributing to neuronal loss pathology. Notably, hippocampal *TMEM14A* expression was significantly reduced in patients with chronic MTLE-HS compared with normal controls, indicating impaired *TMEM14A* function. This hypothesized mechanism aligns well with the distinctive features of MTLE-HS, including the history of precipitating injuries and the histological pattern of neuronal loss, distinguishing it from other forms of focal epilepsy such as focal cortical dysplasia.

Our study has the following limitations. First, the sample size for both cases and controls were relatively small because both groups were limited to focal epilepsy patients. Consequently, the *p*-values (*p* < 1 × 10^−4^) were not as significant as those observed in previous GWASs. To address this limitation and support the biological relevance of our findings, we implemented an integrative validation approach incorporating sQTL/eQTL analyses and transcriptomic profiling. Second, while we identified significant abnormal splicing events involving *TMEM14A* in hippocampal tissue, our study did not investigate the downstream biological consequences of this splicing. Further mechanistic studies will be needed to clarify the functional impact of this abnormal transcript. Third, although the findings indicated a potential link between *TMEM14A* and MTLE-HS, direct evidence for a causal relationship is lacking. Future studies using gene-editing approaches to manipulate *TMEM14A* expression and observe MTLE-HS-related phenotypes in animal models are essential to establish causality.

## 5. Conclusions

Here, we identified rs6924849 as a novel genetic variant associated with MTLE-HS in a Korean population using a GWAS. Integrative genomic and transcriptomic analyses, including sQTL and transcriptomic expression comparison, further elucidated the association between rs6924849, *TMEM14A*, and MTLE-HS. We propose that individuals carrying rs6924849 may exhibit the dysregulated splicing of *TMEM14A* in the hippocampus, leading to the impaired regulation of apoptosis. Combined with initial precipitating injuries, this genetic predisposition may result in unregulated neuronal death, contributing to HS. These findings enhance the understanding of the pathogenesis of MTLE-HS and provide a foundation for the development of novel therapeutic strategies targeting *TMEM14A*.

## Figures and Tables

**Table 1:** Clinical characteristics of focal epilepsy patients with and without HS.

	HS Group (N = 52)	Non-HS Group (N = 105)	*p*-Value
Age, years, mean ± SD	36.5 ± 9.9	35.4 ± 10.7	0.496
Sex, female percentage	51.9	45.7	0.573
Etiology, *n* (%)	-	-	<0.001
Symptomatic	52 (100.0)	45 (42.9)	-
Cryptogenic	0 (0.0)	60 (57.1)	-
Seizure type, *n* (%)	-	-	-
Focal seizure with awareness	1 (1.9)	44 (41.9)	<0.001
Focal seizure with impaired awareness	52 (100.0)	76 (72.4)	<0.001
Focal to bilateral tonic–clonic	6 (11.5)	44 (41.9)	<0.001
Epileptogenic zone, *n* (%)			
Frontal	0 (0.0)	30 (28.6)	-
Temporal	52 (100.0)	37 (35.2)	-
Parietal	0 (0.0)	8 (7.6)	-
Occipital	0 (0.0)	6 (5.7)	-
Unknown	0 (0.0)	24 (22.9)	-

Continuous variables were compared using a two-sample Student’s *t*-test, while nominal variables were analyzed using the chi-square test or Fisher’s exact test. Abbreviations: HS, hippocampal sclerosis; SD, standard deviation.

**Table 2:** Overview of 5 potential candidate SNPs.

SNP	Chr	Position GRCh37	Position GRCh38	Nearest Gene	Region	MAF					Alleles (Major/ Minor)	OR (95% CI)	*p*-Value
						Case	Control	Korea	Japan	Europe			
rs1436751	1	65064340	64598657	*CACHD1*	intron	0.44	0.2048	0.2611	0.1996	0.2684	A/G	3.194 (1.812, 5.63)	0.000059
rs452930	6	94922837	94213119			0.5577	0.3317	0.4273	0.4113	0.3191	C/T	3.735 (1.962, 7.109)	0.000059
rs11696024	2	19812104	19612343			0.3942	0.1810	0.2867	0.2784	0.8340	G/A	3.473 (1.889, 6.384)	0.000061
rs6924849	6	52567028	52702230	*TMEM14A*	downstream	0.4423	0.1857	0.1938	0.1923	0.0467	T/C	2.685 (1.656, 4.353)	0.000061
rs17219864	7	20773567	20733944	*ABCB5*	intron	0.3558	0.1619	0.1983	0.1768	0.3529	G/T	3.549 (1.878, 6.708)	0.000096

This table provides a summary of five SNPs with an allelic χ^2^ *p*-value < 1 × 10^−4^ identified from the genotype data. Allele frequencies for the Korean, Japanese, and European populations were obtained from the Korea Centers for Disease Control and Prevention (KCDC), 14K JPN, and the 1000 Genomes Project Phase 3 in Database of Single Nucleotide Polymorphisms (dbSNP), respectively. Abbreviations: SNP, single-nucleotide polymorphism; Chr, chromosome; MAF, minor allele frequency; OR, odds ratio; CI, confidence interval.

## Data Availability

The summary statistics for all SNPs included in this GWAS are available through the Figshare repository with the following digital object identifier (DOI): https://doi.org/10.6084/m9.figshare.27948009.v1.

## Supplementary Materials

The following supporting information can be downloaded at https://www.mdpi.com/article/10.3390/jcm14113810/s1, Figure S1: Quantile–quantile plot of GWAS data after quality control; Figure S2: Manhattan plot of phenotype data for rs6924849; Figure S3: Expression quantitative trait locus (eQTL) analysis for rs6924849; Figure S4: Bulk tissue gene expression for *TMEM14A*; Table S1: Summary of 127 SNPs with allelic χ^2^ *p*-value < 1×10^−3^; Table S2: Predicted human and mouse phenotypes for *TMEM14A* from the ARCHS4 database.

## Abbreviations

The following abbreviations are used in this manuscript:

HS	hippocampal sclerosis
SNPs	single-nucleotide polymorphisms
MTLE	mesial temporal lobe epilepsy
sQTL	splicing quantitative trait loci
eQTL	expression quantitative trait loci
GTEx	Genotype-Tissue Expression
NES	normalized effect size
LD	linkage disequilibrium
EAS	East Asian
EUR	European
