# Supplementary material for: TMEM14A Gene Affects Hippocampal Sclerosis in Mesial Temporal Lobe Epilepsy
Source: J Clin Med. 2025 May 29;14(11):3810. doi: 10.3390/jcm14113810 (PMC12156207; doi:10.3390/jcm14113810)
Supplement: Supplementary file 1 [file jcm-14-03810-s001.zip › JCM_FigS_legend.docx]

Fig S1. Quantile-quantile plot of GWAS data after quality control.

This quantile-quantile plot depicts GWAS data from 52 epilepsy patients with HS and 105 epilepsy patients without HS. The P-values were obtained using an allelic association test. Black dots represent SNP results after applying quality control filters to the data, while the red line indicates y=x.
Abbreviations: GWAS, genome-wide association study; HS, hippocampal sclerosis; SNP, single-nucleotide polymorphism.

Fig S2. Manhattan plot of phenotype data for rs6924849.

This plot visualizes the phenotypes associated with rs6924849, categorized into respective groups. The Y-axis represents the −log_10_(P-value) for each phenotype. The P value and Beta for the top-ranked phenotype within the neurological category are highlighted with a black circle. The plot was generated using data available on the PheWAS website: https://pheweb.org/UKB-SAIGE/.

Abbreviations: NOS, not otherwise specified

Fig S3. Expression quantitative trait loci (eQTL) analysis for rs6924849.

Normalized eQTL effect size of rs6924849 across 47 tissues from the GTEx v8 dataset, with brain tissues highlighted in red. The –log_10_(P-value) is represented by the size of each point. Error bars indicate the 95% confidence intervals of the effect size.

Abbreviations: GTEx, Genotype-Tissue Expression; eQTL, expression quantitative trait loci

Fig S4. Bulk tissue gene expression for TMEM14A.

The bulk tissue gene expression (TPM) for TMEM14A across tissues from the GTEx v8 dataset, with brain tissues colored in yellow. This violin plot was obtained from the GTEx portal website.

Abbreviations: TPM, transcripts per kilobase million; GTEx, Genotype-Tissue Expression
