# Supplementary material for: TMEM14A Gene Affects Hippocampal Sclerosis in Mesial Temporal Lobe Epilepsy
Source: J Clin Med. 2025 May 29;14(11):3810. doi: 10.3390/jcm14113810 (PMC12156207; doi:10.3390/jcm14113810)
Supplement: Supplementary file 1 [file jcm-14-03810-s001.zip › JCM_TableS1.docx]

**Table S1. Summary of 127 SNPs with an allelic χ² P value < 1×10^-3^.**

This table summarizes 127 SNPs with an allelic χ² P-value < 1 × 10^-3^ identified from the genotype data.

Abbreviations: SNP, single-nucleotide polymorphism; Chr, chromosome; OR, odds ratio; CI, confidence interval; 3′-UTR, three prime untranslated region

| **SNP** | **Chr** | **Position** | **Nearest Gene** | **Region** | **OR (95% CI)** | **P-value** | |
| --- | --- | --- | --- | --- | --- | --- | --- |
| rs1436751 | 1 | 65064340 | CACHD1 | intron | 3.194(1.812,5.63) | 6.E-05 |  |
| rs452930 | 6 | 94922837 |  |  | 3.735(1.962,7.109) | 6.E-05 |  |
| rs11696024 | 2 | 19812104 |  |  | 3.473(1.889,6.384) | 6.E-05 |  |
| rs6924849 | 6 | 52567028 | TMEM14A,GSTA7P | intron,downstream | 2.685(1.656,4.353) | 6.E-05 |  |
| rs17219864 | 7 | 20773567 | ABCB5 | intron | 3.549(1.878,6.708) | 1.E-04 |  |
| rs12407667 | 1 | 65083900 | CACHD1 | intron | 3.069(1.743,5.405) | 1.E-04 |  |
| rs10261664 | 7 | 82713446 | PCLO | intron | 0.3135(0.1738,0.5656) | 1.E-04 |  |
| rs9533329 | 13 | 43549782 | EPSTI1 | intron | 3.222(1.775,5.85) | 1.E-04 |  |
| rs6859754 | 5 | 168649662 | SLIT3 | intron | 5.382(2.269,12.77) | 1.E-04 |  |
| rs2554655 | 8 | 3791547 | CSMD1 | intron | 0.2808(0.1458,0.5409) | 1.E-04 |  |
| rs2302803 | 17 | 35634023 | ACACA | intron | 0.3423(0.1961,0.5974) | 2.E-04 |  |
| rs6459378 | 6 | 14828116 |  |  | 2.782(1.635,4.735) | 2.E-04 |  |
| rs17265801 | 4 | 91911048 | CCSER1 | intron | 3.549(1.833,6.872) | 2.E-04 |  |
| rs2754186 | 16 | 1905763 | LINC00254,MEIOB | intron | 2.63(1.583,4.37) | 2.E-04 |  |
| rs6831193 | 4 | 100908585 | DDIT4L,LOC256880 | intron,downstream | 2.943(1.667,5.196) | 2.E-04 |  |
| rs7983067 | 13 | 44911556 |  |  | 0.3365(0.1889,0.5994) | 2.E-04 |  |
| rs17170960 | 7 | 148038227 | CNTNAP2,MIR548T | intron | 0.2843(0.1458,0.5543) | 2.E-04 |  |
| rs6958824 | 7 | 148038351 | CNTNAP2,MIR548T | intron | 0.2843(0.1458,0.5543) | 2.E-04 |  |
| rs7971796 | 12 | 47463425 |  |  | 0.3038(0.1614,0.5719) | 2.E-04 |  |
| rs1155593 | 8 | 73402635 |  |  | 2.614(1.568,4.356) | 2.E-04 |  |
| rs4358083 | 2 | 19323073 | NT5C1B,MIR4757 | intron,upstream | 0.3343(0.1867,0.5989) | 2.E-04 |  |
| rs17433352 | 5 | 32049787 | PDZD2 | intron | 3.789(1.865,7.698) | 2.E-04 |  |
| rs6790153 | 3 | 155088878 |  |  | 2.614(1.567,4.361) | 2.E-04 |  |
| rs11562068 | 7 | 82708581 | PCLO | intron | 0.3365(0.1883,0.6014) | 2.E-04 |  |
| rs2190043 | 7 | 82709023 | PCLO | intron | 0.3365(0.1883,0.6014) | 2.E-04 |  |
| rs7784368 | 7 | 82713653 | PCLO | intron | 0.2148(0.09431,0.4892) | 2.E-04 |  |
| rs2975966 | 8 | 56379919 | XKR4 | intron | 0.3709(0.2179,0.6315) | 3.E-04 |  |
| rs2929049 | 8 | 56364520 | XKR4,SBF1P1 | intron,exon | 0.3709(0.2179,0.6315) | 3.E-04 |  |
| rs9567416 | 13 | 44908741 |  |  | 0.2952(0.1534,0.5683) | 3.E-04 |  |
| rs1506678 | 12 | 45249492 | NELL2 | intron | 0.3591(0.2063,0.6252) | 3.E-04 |  |
| rs6693972 | 1 | 65096465 | CACHD1 | intron | 2.687(1.567,4.607) | 3.E-04 |  |
| rs10408898 | 19 | 57379704 |  |  | 0.3627(0.2085,0.631) | 3.E-04 |  |
| rs3210458 | 3 | 141011630 | PXYLP1 | synon,intron | 4.038(1.883,8.658) | 3.E-04 |  |
| rs11941799 | 4 | 100767903 | DAPP1 | intron | 2.752(1.583,4.786) | 3.E-04 |  |
| rs17642476 | 17 | 43656380 |  |  | 8.638(2.651,28.14) | 3.E-04 |  |
| rs11096609 | 2 | 19837614 |  |  | 3.055(1.657,5.634) | 3.E-04 |  |
| rs2013613 | 2 | 19811277 |  |  | 3.055(1.657,5.634) | 3.E-04 |  |
| rs10211368 | 2 | 19811982 |  |  | 3.055(1.657,5.634) | 3.E-04 |  |
| rs10186907 | 2 | 19797838 |  |  | 2.91(1.617,5.237) | 4.E-04 |  |
| rs7806858 | 7 | 82713940 | PCLO | intron | 0.2391(0.1088,0.5255) | 4.E-04 |  |
| rs12707545 | 7 | 82714918 | PCLO | intron | 0.2391(0.1088,0.5255) | 4.E-04 |  |
| rs4975166 | 4 | 80127066 | LINC01088 | intron | 3.976(1.86,8.499) | 4.E-04 |  |
| rs11505922 | 7 | 114010651 | FOXP2 | intron | 3.322(1.715,6.435) | 4.E-04 |  |
| rs9525944 | 13 | 44929095 |  |  | 0.3008(0.155,0.5835) | 4.E-04 |  |
| rs13429512 | 2 | 19841370 |  |  | 3.021(1.638,5.572) | 4.E-04 |  |
| rs4909376 | 8 | 138604358 |  |  | 0.3445(0.1908,0.622) | 4.E-04 |  |
| rs28368958 | 8 | 138603731 |  |  | 0.3445(0.1908,0.622) | 4.E-04 |  |
| rs4322801 | 2 | 19319834 | NT5C1B,MIR4757 | intron,upstream | 0.3616(0.2056,0.6359) | 4.E-04 |  |
| rs10494291 | 1 | 153182291 |  |  | 4.455(1.943,10.22) | 4.E-04 |  |
| rs12758356 | 1 | 65091716 | CACHD1 | intron | 2.637(1.538,4.522) | 4.E-04 |  |
| rs1783222 | 11 | 116037721 |  |  | 2.654(1.542,4.568) | 4.E-04 |  |
| rs1624049 | 11 | 116037231 |  |  | 2.654(1.542,4.568) | 4.E-04 |  |
| rs1356873 | 2 | 188636921 | TFPI,GULP1 | intron,upstream | 3.747(1.795,7.821) | 4.E-04 |  |
| rs17029573 | 4 | 100760069 | DAPP1 | intron | 2.697(1.551,4.69) | 4.E-04 |  |
| rs4128628 | 5 | 179038877 |  |  | 3.56(1.752,7.231) | 4.E-04 |  |
| rs11881055 | 19 | 57375063 |  |  | 0.3735(0.2155,0.6473) | 4.E-04 |  |
| rs6027337 | 20 | 58712478 | MIR646HG,LOC729296 | intron,upstream,downstream | 2.395(1.47,3.902) | 5.E-04 |  |
| rs950541 | 15 | 89754954 | RLBP1 | intron | 0.253(0.1173,0.5457) | 5.E-04 |  |
| rs10824362 | 10 | 77847107 | C10orf11 | intron | 0.3797(0.2207,0.6532) | 5.E-04 |  |
| rs2680398 | 17 | 35588925 | ACACA | intron | 0.3771(0.218,0.6522) | 5.E-04 |  |
| rs372827 | 6 | 14846678 |  |  | 2.504(1.495,4.193) | 5.E-04 |  |
| rs2004114 | 2 | 19811249 |  |  | 2.952(1.606,5.427) | 5.E-04 |  |
| rs17035306 | 2 | 68559450 |  |  | 2.604(1.52,4.46) | 5.E-04 |  |
| rs720935 | 21 | 33084257 | SCAF4 | intron | 2.604(1.52,4.46) | 5.E-04 |  |
| rs7162271 | 15 | 98800468 | FAM169B,LOC101927332 | intron,downstream,upstream | 2.392(1.462,3.913) | 5.E-04 |  |
| rs1837993 | 15 | 98801086 | FAM169B,LOC101927332 | intron,downstream,upstream | 2.392(1.462,3.913) | 5.E-04 |  |
| rs1835326 | 2 | 115281105 | DPP10 | intron | 2.964(1.605,5.475) | 5.E-04 |  |
| rs6033214 | 20 | 11724001 |  |  | 0.3829(0.2226,0.6587) | 5.E-04 |  |
| rs8000903 | 13 | 44960248 | SERP2 | intron | 0.307(0.1573,0.5991) | 5.E-04 |  |
| rs7326226 | 13 | 44912736 |  |  | 0.3426(0.1868,0.6284) | 5.E-04 |  |
| rs35902115 | 17 | 6158786 |  |  | 3.326(1.683,6.571) | 5.E-04 |  |
| rs7818823 | 8 | 59580354 |  |  | 3.186(1.652,6.142) | 5.E-04 |  |
| rs2649851 | 3 | 140362036 |  |  | 0.3678(0.2082,0.6497) | 6.E-04 |  |
| rs5749509 | 22 | 33180076 | SYN3 | intron | 3.699(1.756,7.792) | 6.E-04 |  |
| rs7337290 | 13 | 112505755 |  |  | 0.3616(0.2026,0.6456) | 6.E-04 |  |
| rs11205222 | 1 | 153182979 |  |  | 4.614(1.929,11.04) | 6.E-04 |  |
| rs1535571 | 10 | 77621818 | C10orf11 | intron | 5.859(2.132,16.1) | 6.E-04 |  |
| rs11795353 | 9 | 34414339 | FAM219A | intron | 2.547(1.492,4.347) | 6.E-04 |  |
| rs7093663 | 10 | 77621520 | C10orf11 | intron | 5.859(2.132,16.1) | 6.E-04 |  |
| rs950540 | 15 | 89754864 | RLBP1 | intron | 0.2734(0.1303,0.5739) | 6.E-04 |  |
| rs9964158 | 18 | 57619528 |  |  | 0.407(0.2432,0.6811) | 6.E-04 |  |
| rs10848821 | 12 | 3298031 | TSPAN9 | intron | 2.911(1.578,5.37) | 6.E-04 |  |
| rs12649267 | 4 | 137256253 | PCDH18,LINC00613 | intron,downstream,upstream | 0.3718(0.2107,0.6559) | 6.E-04 |  |
| rs3733463 | 4 | 100757048 | DAPP1 | intron | 2.655(1.516,4.65) | 6.E-04 |  |
| rs943051 | 13 | 25241241 |  |  | 0.3077(0.1563,0.6058) | 6.E-04 |  |
| rs4694087 | 4 | 71919167 |  |  | 3.432(1.689,6.975) | 7.E-04 |  |
| rs792385 | 17 | 55430101 | MSI2 | intron | 0.3317(0.1758,0.6258) | 7.E-04 |  |
| rs2407110 | 5 | 121592698 |  |  | 3.216(1.642,6.3) | 7.E-04 |  |
| rs1961328 | 11 | 116040267 |  |  | 2.496(1.474,4.227) | 7.E-04 |  |
| rs7127767 | 11 | 5702099 | TRIM5 | intron | 2.326(1.43,3.782) | 7.E-04 |  |
| rs17655486 | 17 | 6160753 |  |  | 3.248(1.647,6.407) | 7.E-04 |  |
| rs1549760 | 7 | 150755839 | SLC4A2 | intron,UTR-5 | 2.569(1.489,4.431) | 7.E-04 |  |
| rs9362482 | 6 | 89036520 | CNR1,LOC101928936 | intron,upstream,downstream | 0.345(0.1865,0.6381) | 7.E-04 |  |
| rs1783221 | 11 | 116035804 |  |  | 2.537(1.481,4.346) | 7.E-04 |  |
| rs730259 | 2 | 115280178 | DPP10 | intron | 2.876(1.56,5.303) | 7.E-04 |  |
| rs1516136 | 2 | 146129223 |  |  | 0.4044(0.239,0.6842) | 7.E-04 |  |
| rs10861455 | 12 | 105933320 |  |  | 2.869(1.555,5.293) | 7.E-04 |  |
| rs2045528 | 11 | 30602310 | MPPED2 | intron | 0.3893(0.2249,0.6738) | 8.E-04 |  |
| rs8003616 | 14 | 52646094 |  |  | 0.3301(0.1727,0.631) | 8.E-04 |  |
| rs12213461 | 6 | 159826155 |  |  | 0.2596(0.1179,0.5716) | 8.E-04 |  |
| rs7592790 | 2 | 151409434 | LOC101929260 | exon | 6.318(2.146,18.6) | 8.E-04 |  |
| rs16967199 | 17 | 66776168 |  |  | 3.184(1.615,6.277) | 8.E-04 |  |
| rs17735410 | 5 | 168627798 | SLIT3 | intron | 4.796(1.913,12.02) | 8.E-04 |  |
| rs17667992 | 5 | 168644410 | SLIT3 | intron | 4.796(1.913,12.02) | 8.E-04 |  |
| rs7715391 | 5 | 179016882 | RUFY1 | intron | 3.385(1.654,6.928) | 8.E-04 |  |
| rs7207687 | 17 | 14152804 |  |  | 0.3952(0.2291,0.6818) | 8.E-04 |  |
| rs12538858 | 7 | 29392533 | CHN2 | intron | 3.155(1.605,6.202) | 9.E-04 |  |
| rs10215935 | 7 | 29394539 | CHN2 | intron | 3.155(1.605,6.202) | 9.E-04 |  |
| rs2688266 | 8 | 3791362 | CSMD1 | intron | 0.3589(0.1964,0.6558) | 9.E-04 |  |
| rs9309413 | 2 | 68570772 |  |  | 5.139(1.96,13.48) | 9.E-04 |  |
| rs1026394 | 4 | 80108181 | LINC01088 | intron | 3.453(1.664,7.167) | 9.E-04 |  |
| rs10494294 | 1 | 153185017 |  |  | 4.099(1.782,9.427) | 9.E-04 |  |
| rs11031762 | 11 | 32407854 |  |  | 3.077(1.585,5.976) | 9.E-04 |  |
| rs6064892 | 20 | 58702400 | MIR646HG,LOC729296 | intron,upstream,downstream | 2.533(1.463,4.385) | 9.E-04 |  |
