# Supplementary material for: TMEM14A Gene Affects Hippocampal Sclerosis in Mesial Temporal Lobe Epilepsy
Source: J Clin Med. 2025 May 29;14(11):3810. doi: 10.3390/jcm14113810 (PMC12156207; doi:10.3390/jcm14113810)
Supplement: Supplementary file 1 [file jcm-14-03810-s001.zip › JCM_TableS2.docx]

**Table S2. Predicted Human and Mouse Phenotypes for TMEM14A from the ARCHS4 Database.**

This table summarizes the predicted human and mouse phenotype gene sets associated with TMEM14A from the ARCHS4 database. Each phenotype is ranked by Z-score, which reflects the correlation between TMEM14A and known members of the corresponding gene set. A higher Z-score indicates a stronger correlation and higher likelihood of membership prediction. Note that TMEM14A is not a confirmed member of all the gene sets listed in this table. The phenotypes related to epilepsy and seizure are highlighted in bold.

Abbreviations: ARCHS4, All RNA-seq and ChIP-seq sample and signature search; HP, Human Phenotype Ontology; MP, Mammalian Phenotype Ontology

|  | Gene Set | Ontology | Z-score |
| --- | --- | --- | --- |
| Predicted Human Phenotypes | **Focal motor seizures** | HP:0011153 | 6.2546 |
|  | Myokymia | HP:0002411 | 4.7465 |
|  | **Epileptic encephalopathy** | HP:0200134 | 4.6506 |
|  | **Atonic seizures** | HP:0010819 | 4.5976 |
|  | Acute necrotizing encephalopathy | HP:0006965 | 4.3620 |
|  | **Focal seizures** | HP:0007359 | 4.0792 |
|  | Neurofibrillary tangles | HP:0002185 | 3.9041 |
|  | Hyperventilation | HP:0002883 | 3.8919 |
|  | Abnormal mitochondria in muscle tissue | HP:0008316 | 3.8856 |
|  | Progressive macrocephaly | HP:0004481 | 3.7475 |
| Predicted Mouse Phenotypes | **Abnormal central pattern** | MP0003880 | 5.4378 |
|  | **Abnormal synaptic plasticity** | MP0004859 | 4.1348 |
|  | **Abnormal synaptic transmission** | MP0003635 | 3.3729 |
|  | Analgesia | MP0004270 | 3.2626 |
|  | Abnormal behavioral response | MP0009745 | 3.2396 |
|  | **Seizures** | MP0002064 | 3.0206 |
|  | Abnormal touch/nociception | MP0001968 | 2.9267 |
|  | Abnormal vocalization | MP0001529 | 2.9240 |
|  | **Muscle twitch** | MP0009046 | 2.7943 |
|  | **Abnormal nervous system** | MP0002272 | 2.7887 |
