# Supplementary figures and images for: TMEM14A Gene Affects Hippocampal Sclerosis in Mesial Temporal Lobe Epilepsy
Source: J Clin Med. 2025 May 29;14(11):3810. doi: 10.3390/jcm14113810 (PMC12156207; doi:10.3390/jcm14113810)

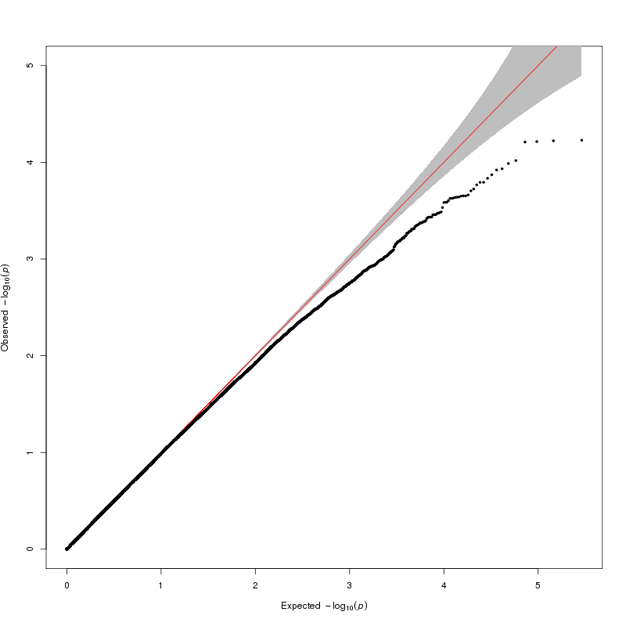

Supplement: Supplementary file 1 [file jcm-14-03810-s001.zip › JCM_FigS1.png]

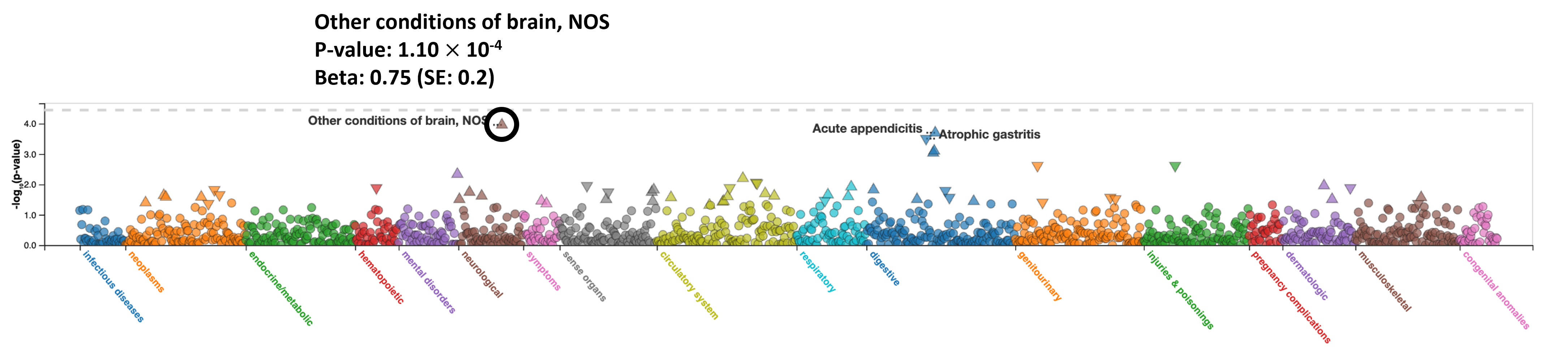

Supplement: Supplementary file 1 [file jcm-14-03810-s001.zip › JCM_FigS2.png]

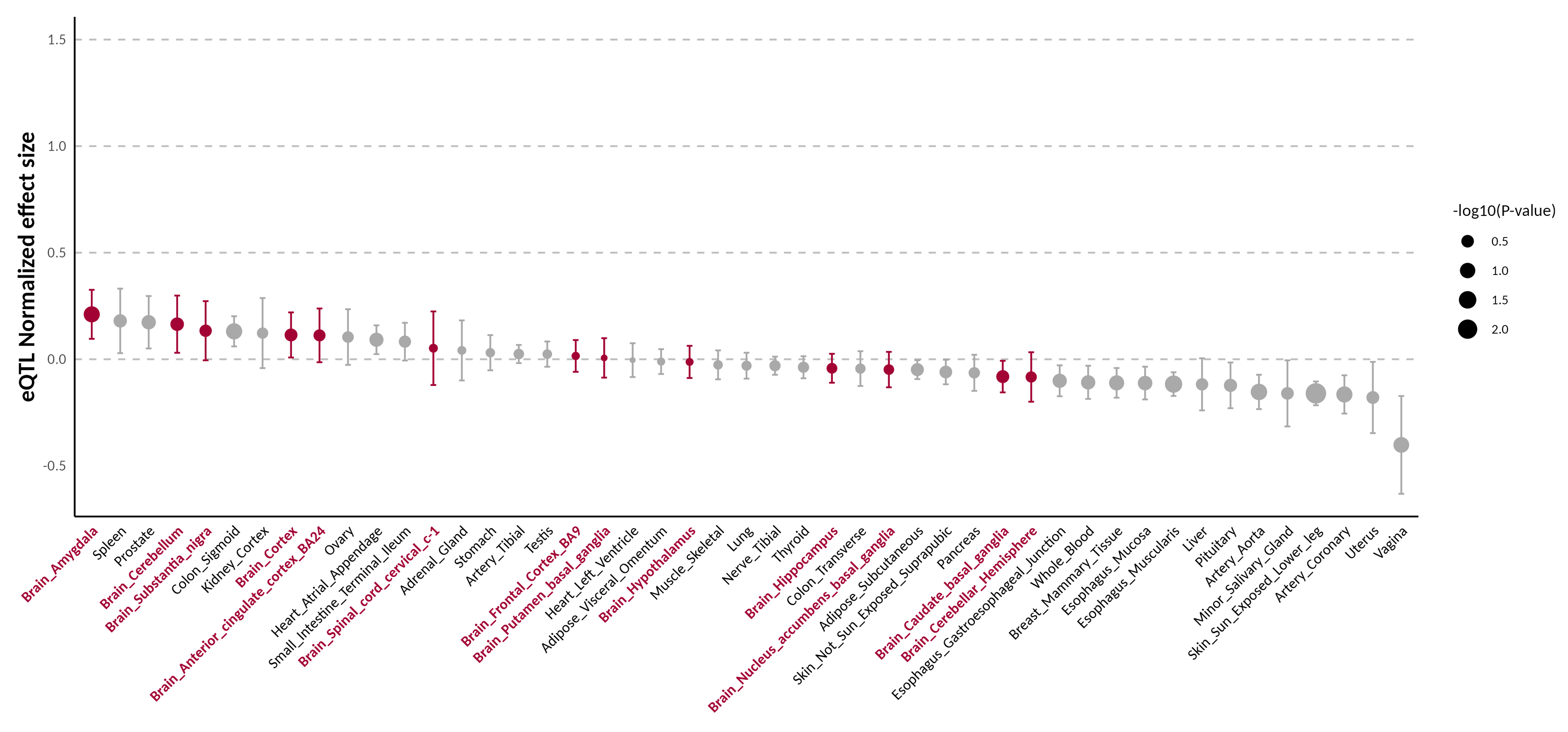

Supplement: Supplementary file 1 [file jcm-14-03810-s001.zip › JCM_FigS3.png]

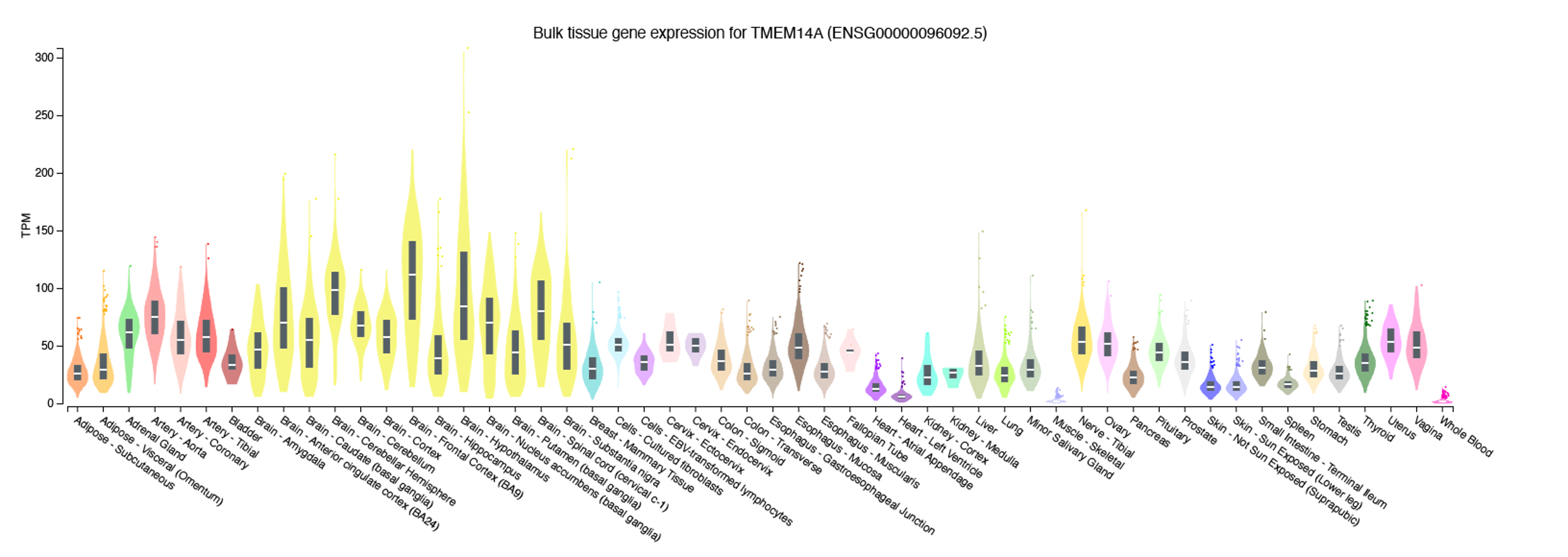

Supplement: Supplementary file 1 [file jcm-14-03810-s001.zip › JCM_FigS4.png]
